# A Variant in Genes of the NPY System as Modifier Factor of Machado-Joseph Disease in the Chinese Population

**DOI:** 10.3389/fnagi.2022.822657

**Published:** 2022-02-03

**Authors:** Dongxue Ding, Zhao Chen, Chunrong Wang, Xiang Tang, Lulu Zhang, Qi Fang, Rong Qiu, Hong Jiang

**Affiliations:** ^1^Department of Neurology, The First Affiliated Hospital of Soochow University, Suzhou, China; ^2^Department of Neurology, Xiangya Hospital, Central South University, Changsha, China; ^3^Department of Pathology, Xiangya Hospital, Central South University, Changsha, China; ^4^School of Information Science and Engineering, Central South University, Changsha, China; ^5^Key Laboratory of Hunan Province in Neurodegenerative Disorders, Central South University, Changsha, China; ^6^National Clinical Research Center for Geriatric Diseases, Central South University, Changsha, China; ^7^Laboratory of Medical Genetics, Central South University, Changsha, China; ^8^School of Basic Medical Science, Central South University, Changsha, China; ^9^Hunan International Scientific and Technological Cooperation Base of Neurodegenerative and Neurogenetic Diseases, Changsha, China

**Keywords:** Machado-Joseph disease, age at onset, *NPY*, *NPY2R*, *NPY5R*, SNPs

## Abstract

Recently, NPY overexpression has been proposed to alleviate motor deficits and neuropathy in Machado-Joseph disease (MJD) mouse models, indicating its neuroprotective role in the pathogenesis of MJD. We aimed to evaluate the association between SNPs in *NPY* and its receptors and the susceptibility of MJD in the Chinese population. Moreover, we investigated whether these SNPs modulate the age at onset (AO) of MJD. In total, 527 MJD patients and 487 healthy controls were enrolled in the study, and four specific selected SNPs (rs16139, rs3037354, rs2234759, and rs11100494) in *NPY* and its receptor genes were genotyped. In this study, the genotypic frequency using the dominant model and the allelic distribution of rs11100494 in *NPY5R* revealed a significant difference between the MJD and control group during the first-stage analysis (*P* = 0.048 and *P* = 0.024, respectively). After we expanded the sample size, significant differences were observed between the two groups using the dominant model in genotypic and allelic distribution (*P* = 0.034, *P* = 0.046, and *P* = 0.016, respectively). No significant differences in genotypic and allelic distribution were found between the MJD and control groups for the other three SNPs. All selected SNPs had no significant effect on the AO of MJD. The association of rs11100494 in the *NPY5R* gene and susceptibility of MJD suggested that the NPY system might be implicated in the pathogenesis of MJD. Our study demonstrated the existence of other genetic modifiers in MJD, along with CAG expansion and known genetic modifier factors, which might lead to a better understanding of MJD pathogenesis.

## Introduction

Machado-Joseph disease (MJD), or spinocerebellar ataxia type 3 (SCA3), is a fatal, autosomal dominantly inherited neurodegenerative disease without curable therapies (Paulson, 2007). It was caused by an abnormal expansion of the CAG repeats in exon 10 of the *ATXN3* gene, leading to an expanded polyglutamine tract within the ataxin3 protein (Kawaguchi et al., 1994). The expanded polyglutamine tract induces insoluble ubiquitin-positive protein aggregation and accumulation in neurons, leading to progressive neurodegeneration in the cerebellum, spinal cord, and substantia nigra (Taroni and DiDonato, 2004). There is a well-established inverse correlation between abnormally expanded CAG repeats and the age at onset (AO) in MJD (Matos et al., 2019). However, the abnormally expanded CAG repeats could only explain 50—70% of AO variability, showing that MJD is modulated by factors other than the expanded CAG repeats only (Tezenas et al., 2014; Chen et al., 2016). Recent studies have reported that in addition to CAG repeats in polyglutamine (polyQ) genes (Franca et al., 2012; Leotti et al., 2021), single nucleotide polymorphisms (SNPs) in crucial genes could also modulate the AO of MJD (Wang et al., 2018; Ding et al., 2019; Mergener et al., 2020), indicating that they could be new important genetic factors affecting the pathogenesis of MJD and composing “missing heritability” in AO of MJD (Ding et al., 2016). To date, the genotypic and/or allelic status of SNPs at approximately 10 genes, including *ATXN3* (Long et al., 2015), *ATXN2* (Ding et al., 2016), *APOE* (Bettencourt et al., 2011; Peng et al., 2014), *MT-ND3* (Chen et al., 2016), *CAST* (Martins et al., 2021), *FAN1* (Mergener et al., 2020), *DNMT3A*, *DNMT3L* (Ding et al., 2019), and *IL6* (Raposo et al., 2017), has been demonstrated to modulate the AO of MJD patients. These genes are involved in mitochondrial function, the calpain-cleavage pathway, DNA repair, DNA methylation, and neuroimmunity, suggesting that the mechanism of MJD pathogenesis is complicated.

Neuropeptide Y (NPY) is an abundantly distributed neuropeptide in the mammalian brain and has been implicated in neuroprotection through inhibiting neuron death and excitotoxicity (Smialowska et al., 2009; Santos-Carvalho et al., 2013), increasing neuronal trophic support (Croce et al., 2013), stimulating the process of autophagy (Aveleira et al., 2015), and regulating transmission between cerebellar interneurons (Dubois et al., 2012). To date, five NPY receptors (NPY1R, NPY2R, NPY4R, NPY5R, and NPY6R) have been found in the mammalian brain; however, NPY6R has not been reported to be functional in the human brain (Diaz-delCastillo et al., 2018). NPY produces neuroprotective effects through NPY2R and NPY5R by alleviating the excitatory neurotoxic effect, inhibiting glutamate receptor overactivity, regulating calcium homeostasis, and attenuating neuroinflammation (Wu and Li, 2005; Farzi et al., 2015). In the central nervous system, NPY has been proven to control and alleviate neurodegeneration in models of Parkinson’s disease (PD) (Decressac et al., 2012), Huntington disease (HD) (Decressac et al., 2010), and Alzheimer’s disease (AD) (Chen et al., 2018). Moreover, SNPs (rs16139, rs3037354, rs2234759, and rs11100494) in *NPY*, *NPY2R*, and *NPY5R* have been found to be associated with AO in HD patients, major depressive disorder, and dyslipidemia (Coletta et al., 2007; Wang et al., 2013; Kloster et al., 2014). Recently, NPY overexpression or intranasal delivery in MJD mouse models have been found to alleviate MJD-associated motor deficits and neuropathy, indicating that NPY has neuroprotective potential in the pathogenesis of MJD (Duarte-Neves et al., 2021, 2015). This study was designed to determine whether NPY and its receptors contribute to the susceptibility of MJD and the variability of AO of this disease. For this purpose, we conducted a case–control study that analyzed the association between selected SNPs in *NPY*, *NPY2R*, and *NPY5R* and MJD. We also investigated whether these SNPs could explain some of the variability of AO in MJD patients.

## Materials and Methods

### Study Subjects

We enrolled 527 patients and 487 healthy controls (300 MJD patients and 300 controls from mainland China in the first stage (Supplementary Table 1), and an additional 227 MJD patients and 187 controls were added (Supplementary Table 2) to validate the obtained positive results) from Hunan, Hubei, Guizhou, Jiangxi, and Jiangsu provinces of China. The patients were consecutively recruited from the Department of Neurology of Xiangya Hospital, Central South University and the Department of Neurology of the First Affiliated Hospital of Soochow University during 2004–2020. A standard clinical neurological examination was performed by at least two experienced neurologists, and the clinical diagnoses were confirmed by molecular examinations. The healthy control subjects were matched to MJD patients by gender and did not have a positive family history of spinocerebellar ataxias (SCAs). Written informed consent was obtained from all subjects before they participated in this study, which was approved by the Ethics Committee of Xiangya Hospital, Central South University and the First Affiliated Hospital of Soochow University.

### SNP Selection and Genotyping

Four SNPs (rs16139, rs3037354, rs2234759, and rs11100494) in *NPY*, *NPY2R*, and *NPY5R* were selected by giving priority to SNPs in the promoter or other regulatory regions and SNPs shown to be associated human diseases. Genomic DNA was extracted from peripheral blood leukocytes via standard phenol–chloroform extraction methods. CAG repeats in *ATXN3* were determined by capillary electrophoresis and DNA sequencing with T-vector cloning. Genotypes of SNPs in the primary 600subjects were examined by matrix-assisted laser desorption/ionization-time-of-flight mass spectrometry via the MassARRAY system (Sequenom). To confirm the matrix-assisted laser desorption/ionization-time-of-flight mass spectrometry results, 30 patients and 30 controls were randomly selected for Sanger sequencing. Genotypes of SNPs in an additional 414 subjects (227 MJD patients and 187 controls) were tested by Sanger sequencing. All primers (Supplementary Table 3) were designed using Primer3^1^.

### Statistical Analysis

For each SNP, the standard goodness-of-fit test was used to test Hardy–Weinberg equilibrium (HWE). The differences in the allele and genotype frequencies between cases and controls were determined using the standard chi-square (χ^2^) test or Fisher tests. The odds ratios (ORs) and associated 95% confidence intervals (95% CIs) were also calculated. Linear regression analysis was performed to determine the effect of each SNP on AO with the logarithmically (decimal) transformed AO as the dependent variable. When introducing each SNP in the linear regression analysis, dominant, codominant/genotypic, and recessive effects were assumed. The determinant coefficient (R^2^) was used to indicate the percentage of the explanation for AO variance via a given model. We analyzed covariance to adjust for the effect of the expanded CAG repeats on AO. Before that, a hypothesis test for regression coefficients was performed to examine the interaction between expanded CAG repeats and genotypes.

All analyses were performed using SPSS Ver23.0 (SPSS Inc., Chicago, IL, United States), and *P* < 0.05 was considered statistically significant.

## Results

### Subject Characteristics

The characteristics of MJD patients and healthy controls are summarized in Table 1. The study included 1,014 subjects, comprising 527 MJD patients [274 males and 253 females; mean age (±SD), 40.24 ± 11.65 years; Table 1] and 487 controls [250 males and 237 females; mean age (±SD), 41.83 ± 18.06 years; Table 1]. There was no significant difference in gnder distribution between MJD patients and controls (χ^2^ = 0.044, *P* = 0.834).

**TABLE 1:** Characteristics of the controls and MJD patients.

Characteristic		MJD patients	Controls
Mean age	Total	40.24 ± 11.65	41.83 ± 18.06
	Female	40.88 ± 11.49	41.42 ± 19.28
	Male	39.65 ± 11.79	42.21 ± 16.86
Age of onset	Total	34.90 ± 10.23	NA
	Female	35.55 ± 10.13	
	Male	34.30 ± 10.30	
CAG (normal)	Total	22.62 ± 6.29	NA
	Female	22.93 ± 6.15	
	Male	22.33 ± 6.41	
CAG (expanded)	Total	74.90 ± 3.55	NA
	Female	74.92 ± 3.48	
	Male	74.89 ± 3.62	

### Association of *NPY*, *NPY R2*, and *NPY R5* SNPs With MJD

The genotypic distribution and allelic frequency for the selected SNPs in the MJD patients and controls are described in Table 2. The genotypic distribution and allelic frequency did not deviate significantly from HWE (*P* > 0.05). In the primary cohort of 300 MJD patients and 300 controls, all genotypes of rs16139 in both MJD patients and controls were “TT” (not shown in Table 2). There were no significant differences in the genotypic distribution and allelic frequency of rs2234759 and rs3037354 between MJD patients and controls (Table 2). In the first stage of analysis, the frequencies of rs11100494 genotypes determined from MJD were 78.33% for CC, 20% for CA, and 1.67% for AA, and in the control group, the frequencies of CC, CA, and AA were 71.33, 25, and 3.67%, respectively (*P* = 0.086). The CC genotype of rs11100494 was significantly higher among the MJD patients than among the control group (78.33 and 71.33%, respectively, *P* = 0.048) and was associated with increased susceptibility of MJD (OR = 1.543, 95% CI: 1.002–2.107). The C allelic frequency showed a significant difference in MJD patients compared with the control group (*P* = 0.024, OR = 1.460, 95% CI: 1.049–2.032). After we expanded the sample size, CC, CA, and AA genotypic distributions were significantly different between the MJD patients and the control group (*P* = 0.046). Moreover, significant differences in CC genotypic and C allelic frequencies between MJD patients and the control group remained (*P* = 0.034, OR = 1.343, 95% CI: 1.022–1.767; *P* = 0.016, OR = 1.347, 95% CI: 1.058–1.715, respectively).

**TABLE 2:** Correlation analysis between genotypes and alleles of selected SNPs and susceptibility of MJD.

Study group	variables																				
	**Genotypic distribution**						**Genotypic distribution (dominant model)**				**Genotypic distribution (recessive model)**							**Allelic frequency**			
**rs2234759**	**AA**		**AG**		**GG**		**AA**		**AG + GG**		**AA + AG**				**GG**			**A**		**G**	
	**N**	**%**	**N**	**%**	**N**	**%**	**N**	**%**	**N**	**%**	**N**	**%**			**N**		**%**	**N**	**%**	**N**	**%**

MJD (*N* = 300)	79	26.33	149	49.67	72	24	79	26.33	221	73.67	228	76			72		24	307	51.17	293	48.83
Control (*N* = 300)	71	23.67	155	51.67	74	24.66	71	23.67	229	76.33	226	75.33			74		24.67	297	49.5	303	50.5
*P* value (χ^2^)	0.751 (0.572)						0.451 (0.569)				0.849 (0.036)							0.564 (0.333)			
OR (95%CI)	–						1.153 (0.796–1.669)				1.037 (0.714–1.506)							1.069 (0.852–1.340)			

**rs3037354**	**TG**		**TG.DEL**		**DEL**		**TG**		**TG.DEL + DEL**		**TG + TG.DEL**				**DEL**			**TG**		**DEL**	
	**N**	**%**	**N**	**%**	**N**	**%**	**N**	**%**	**N**	**%**	**N**		**%**		**N**		**%**	**N**	**%**	**N**	**%**

MJD (*N* = 300)	135	45	138	46	27	9	135	45	165	55	273		91		27		9	408	68	192	32
Control (*N* = 300)	134	44.67	125	41.67	41	13.66	134	44.67	166	55.33	259		86.33		41		13.67	393	65.5	207	34.5
*P* value (χ^2^)	0.171 (3.529)						0.935 (0.007)				0.071 (3.251)							0.358 (0.845)			
OR (95%CI)	–						1.014 (0.735–1.398)				1.601 (0.957–2.678)							1.119 (0.880–1.423)			

**rs11100494**	**CC**		**CA**		**AA**		**CC**		**CA + AA**		**CC + CA**				**AA**			**C**		**A**	
	**N**	**%**	**N**	**%**	**N**	**%**	**N**	**%**	**N**	**%**	**N**		**%**		**N**		**%**	**N**	**%**	**N**	**%**

MJD (*N* = 300)	235	78.33	60	20	5	1.67	235	78.33	65	21.67	295		98.33		5		1.67	530	88.33	70	11.67
Control (*N* = 300)	214	71.33	75	25	11	3.67	214	71.33	86	28.67	289		96.33		11		3.67	503	83.83	97	16.17
*P* value (χ^2^)	0.086 (4.899)						**0.048 (3.903)**				0.128 (2.312)							**0.024 (5.071)**			
OR (95%CI)							**1.453 (1.002–2.107)**				2.246 (0.771–6.544)							**1.460 (1.049–2.032)**			

	**N**	**%**	**N**	**%**	**N**	**%**	**N**	**%**	**N**	**%**	**N**	**%**		**N**		**%**		**N**	**%**	**N**	**%**

MJD (*N* = 527)	393	74.57	125	23.72	9	1.71	393	74.57	134	25.43	518	98.29		9		1.71		911		143	
Control (*N* = 487)	334	68.58	136	27.93	17	3.49	334	68.58	86	31.42	470	96.51		17		3.49		804		170	
*P* value (χ^2^)	**0.046 (6.145)**						**0.034 (4.475)**				0.073 (3.221)							**0.016 (5.858)**			
OR (95%CI)	–						**1.343 (1.022–1.767)**				2.082 (0.919–4.715)							**1.347 (1.058–1.715)**			

### Association of *NPY*, *NPY 2R*, and *NPY5R* SNPs With the AO of MJD

**FIGURE 1:**
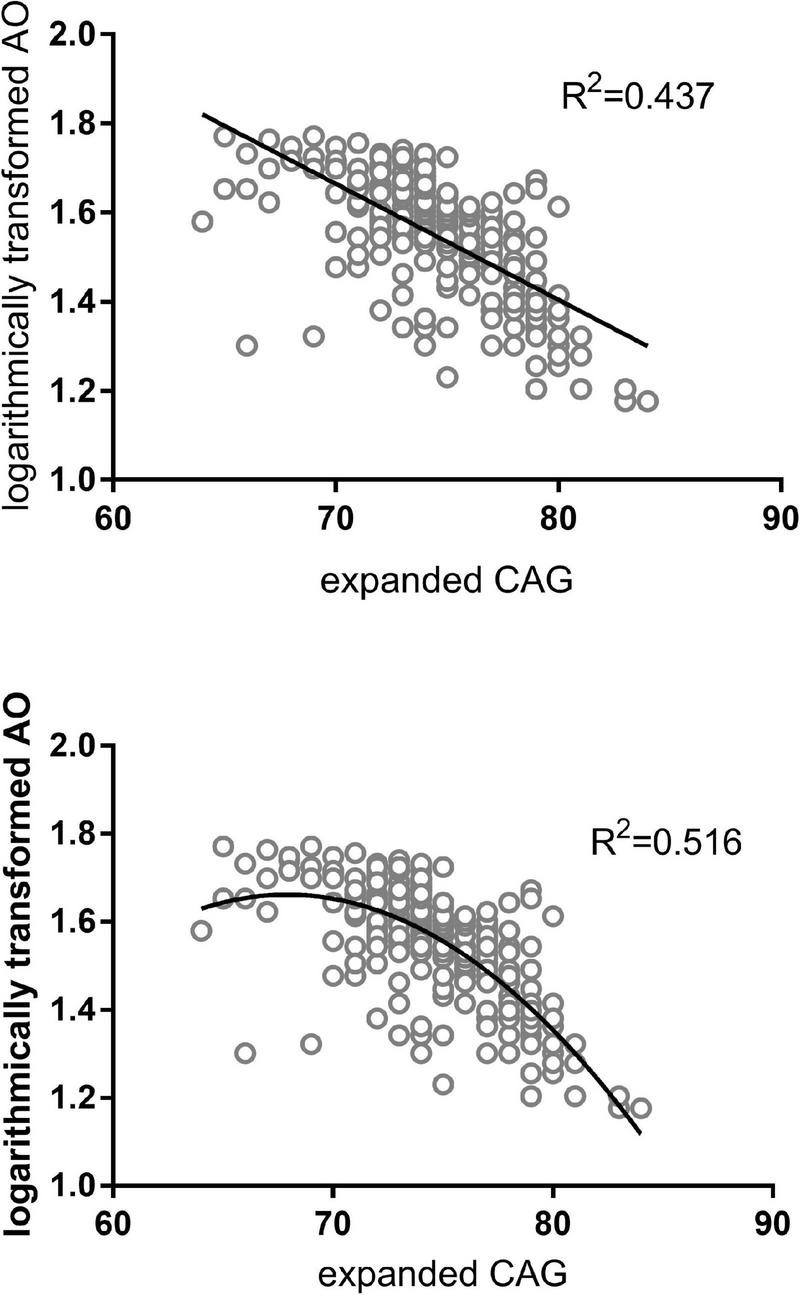
AO attributed to abnormal CAG repeats in *ATXN3* of MJD patients. The figures on the above and below are regression analyses in linear and quadratic models, respectively. The X-axis denotes the expanded CAG repeat length, and the Y-axis indicates the logarithmically transformed AO. AO of MJD patients was inversely correlated with the length of expanded CAG repeats in the *ATXN3* gene.

We inspected the residuals to verify the validity of the linear regression model, and an extreme outlier with CAG repeats of 51 was eliminated in the primary 300-patient cohort. Analyzing the effect of the abnormally expanded CAG repeat in *ATXN3* itself on ln(AO), R^2^ reaches values of 0.437 and 0.516 in linear and quadric models, respectively (Figure 1). Because of the additional increase of 7.9% in R^2^, the quadric model was used for the following analysis. As shown in Table 3, after inclusion of each of the four SNPs, the R^2^ value was raised to different degrees or not raised. However, after adjusting for the expanded CAG repeats in *ATXN3*, there were no significant differences among different genotypes of rs11100494 in the *NPY5R* gene (Table 4) in the analysis of covariance. In the replication stage, another 2 extreme outliers with CAG repeats of 55 and 86 were eliminated. The results of covariance analysis of rs11100494 showed no essential change after we enrolled the additional 227 MJD patients into the cohort (Table 4).

**TABLE 3:** Regression analysis of SNPs in *NPY* and receptor genes in AO of MJD.

Group	Number of patients	R^2^	Δ R^2^	*P-*value
MJD (CAG 65∼84)	299	0.516	–	<0.001
**rs3037354**				
Dominant model	272/27	0.517	0.001	0.615
Codominant/genotypic model	135/137/27	0.517	0.001	0.867
Recessive model	135/164	0.516	–	0.976
**rs2234759**				
Dominant model	227/72	0.522	0.006	0.081
Codominant/genotypic model	79/148/72	0.526	0.010	0.126
Recessive model	79/220	0.517	0.001	0.695
**rs 11100494**				
Dominant model	294/5	0.516	–	0.685
Codominant/genotypic model	234/60/5	0.517	0.001	0.831
Recessive model	234/65	0.517	0.001	0.384
**rs 11100494 (expanded sample)**	524	0.572	–	<0.001
Dominant model	515/9	0.572	–	0.606
Codominant/genotypic model	390/125/9	0.572	–	0.701
Recessive model	390/134	0.572	–	0.594

*Delta (Δ) R^2^ quantifies the relative improvement of the regression model when the SNP genotypes are considered CAG repeats.*

**TABLE 4:** AO differences among different genotypes of rs11100494 in the *NPY5R* gene after adjusting for the effect of expanded CAG repeats.

Study group	Genotype	AO of MJD patients (*N* = 299)	AO of MJD patients (*N* = 524)
Genotypic distribution	CC	36.25 ± 9.92	35.14 ± 10.20
	CA	35.45 ± 10.06	34.43 ± 10.20
	AA	38.60 ± 5.86	34.00 ± 10.98
*P* value		0.814	0.694
Genotypic distribution (dominant model)	CC	36.25 ± 9.92	35.14 ± 10.20
	CA + AA	35.69 ± 9.80	34.40 ± 10.22
*P* value		0.871	0.743

## Discussion

Machado-Joseph disease is the most common type of autosomal dominant ataxia worldwide, and this disease accounts for approximately 62.64% of cases in China (Paulson, 2012; Chen et al., 2018). Although the genetic cause of MJD, abnormal CAG expansion in *ATXN3*, has been clearly defined for many years (Takiyama et al., 1993; Kawaguchi et al., 1994), the pathogenesis mechanisms of MJD have not been fully elucidated. To date, several different pathways have been identified to be involved in the pathogenesis of MJD: RNA toxicity (Nalavade et al., 2013), abnormal protein aggregation (Seidel et al., 2012), dysregulation of transcription (Raposo et al., 2015), proteolytic cleavage (Weber et al., 2017), post-translational modification (Wan et al., 2018), mitochondrial dysfunction (Ramos et al., 2015), calcium signaling dysregulation (Chen et al., 2008), and damage of neuronal homeostasis (Cunha-Santos et al., 2016).

NPY, widely expressed in the central nervous system (CNS), has been implicated in neurogenesis and neuroprotection, playing a crucial role in maintaining neuronal homeostasis (Vezzani et al., 1999). The functions in neurogenesis and neuroprotection are performed by binding to different G-coupled NPY receptors distributed in different organs (Pedrazzini et al., 2003). To date, the NPY system has been found to play a potential role and to be a therapeutic target in many neurodegenerative diseases, such as AD, PD, and HD (Decressac et al., 2010, 2012; Croce et al., 2013). Recently, Duarte-Neves et al. (2015) found that NPY overexpression alleviated motor coordination and balance disability, prevented mutant ataxin3-induced immune activity increase, increased BDNF levels, and reduced IL-6 levels in MJD mouse models. Their results indicate the neuroprotective role of NPY in the pathogenesis of MJD.

This study is the first extensive examination of the association between variations in *NPY*, *NPY2R*, *NPY5R* genes and the pathogenesis and AO of MJD. In the present study, we provide evidence that variation in the *NPY5R* gene is associated with susceptibility to MJD. The genotypic distribution and allelic frequency of rs11100494 were significantly different between MJD patients and the control group. The CC genotypic and C allelic frequencies were significantly higher in MJD patients than in the control group, indicating that they were genetic modifier factors of MJD. To date, this SNP has been demonstrated to be associated with plasma TG levels and HDL concentrations in a Mexican-American dyslipidemia cohort (Coletta et al., 2007) and the phenotype of alcohol withdrawal with seizures (Wetherill et al., 2008). It was proven for the first time to be associated with neurodegenerative disease. As mentioned above, NPY could alleviate the excitatory neurotoxic effect, inhibit glutamate receptor overactivity, regulate calcium homeostasis, and attenuate neuroinflammation by binding to NPY2R and NPY5R in the CNS (Wu and Li, 2005; Farzi et al., 2015). Moreover, IL-6 levels were reduced in an MJD mouse model with NPY overexpression (Duarte-Neves et al., 2015), and a variant in *IL6* was associated with the AO of MJD patients (Raposo et al., 2017). We speculated that rs11100494 might modulate the susceptibility of MJD by influencing the levels of interleukins and the process of neuroinflammation. However, the mechanism remains to be fully elucidated in the future.

In our data, all the genotypes of rs16139 in both MJD patients and controls were “TT”, which is different from some previously reported results (MAF = 0.019 in major depression) (Wang et al., 2013; Kloster et al., 2014). Therefore, this SNP was not included in the next analysis. rs3037354 of the *NPY* gene and rs2234759 of the *NPY2R* gene were previously demonstrated to be associated with the pathogenesis of HD (Kloster et al., 2014). However, no significant differences were observed between MJD patients and controls in our data. This result may be explained by the different distribution of the variations in various racial groups and different diseases. For all SNPs analyzed in our study, no differences in AO were found among their different genotypes and alleles. Due to the incompleteness of the information provided by single SNPs, the genetic association conclusion drawn by individual SNP data analysis may not be definite. Negative results from a specific SNP cannot rule out the possible association of diseases with candidate genes. Given the neuroprotective role of *NPY* and its receptors in neurodegenerative disease (Decressac and Barker, 2012; Decressac et al., 2012; Geloso et al., 2015), exploring other functional SNPs or the whole exons of these genes in MJD patients is suggested before a definite conclusion is reached.

In summary, using a case–control study and an extended analysis in MJD patients, we found that the genotypic distribution and allelic frequency of the rs11100494 polymorphism in the *NPY5R* gene were significantly different between MJD patients and the control group. This result indicates that sequence variations in the NPY system might be associated with the pathogenesis of MJD. Therefore, the NPY system could be of interest in MJD as a possible therapeutic agent, and detection of SNPs in a larger sample should be performed in the future to confirm the results and find other “missing heritability” in MJD.

## Data Availability Statement

The original contributions presented in the study are included in the article/Supplementary material, further inquiries can be directed to the corresponding author.

## Ethics Statement

The studies involving human participants were reviewed and approved by the Ethics Committee of the First Affiliated Hospital of Soochow University; the Ethics Committee of Xiangya Hospital, Central South University. Written informed consent to participate in this study was provided by the participants’ legal guardian/next of kin.

## Author Contributions

DD conceived the research, analyzed the data, and wrote the manuscript. CW and ZC helped in data acquisition, analysis, and interpretation of the data. XT and LZ were involved in data acquisition. QF was involved in the study design. RQ helped with funding and was involved in the study design. HJ helped with funding and was involved in the study design, conceptualization, data acquisition, technical and material support, and critical revision of the manuscript. All authors contributed to the article and approved the submitted version.

## Conflict of Interest

The authors declare that the research was conducted in the absence of any commercial or financial relationships that could be construed as a potential conflict of interest.

## Publisher’s Note

All claims expressed in this article are solely those of the authors and do not necessarily represent those of their affiliated organizations, or those of the publisher, the editors and the reviewers. Any product that may be evaluated in this article, or claim that may be made by its manufacturer, is not guaranteed or endorsed by the publisher.

## Footnotes

1
http://bioinfo.ut.ee/primer3-0.4.0/
