# Supplementary material for: A Variant in Genes of the NPY System as Modifier Factor of Machado-Joseph Disease in the Chinese Population
Source: Front Aging Neurosci. 2022 Feb 3;14:822657. doi: 10.3389/fnagi.2022.822657 (PMC8851415; doi:10.3389/fnagi.2022.822657)
Supplement: Supplementary file 3 [file Table_3.DOCX]

Supplement Table 3 The primers used in this study

| Genes | SNPs | Primers | | Fragment length |
| --- | --- | --- | --- | --- |
|  |  | Forward | Reverse |  |
| NPY | rs16139 | CTGGTGTGCAGGCACTGG | CCTGCAGATGCTAGGTAACAA | 248bp |
| NPY | rs3037354 | CAGAACCCACATTCTCAACG | CTATTCCCCCAGTTTGCTGA | 400bp |
| NPY2R | rs2234759 | CCAGCCTGAGGTCTCCTTC | CTACCTCCCTTCCCTTCCAC | 399bp |
| NPY5R | rs11100494 | AGCCTGCTAGTCTGTCAGTG | TGCCTACCCATCTACCTTGT | 394bp |
